# Randomized controlled trial assessing the impact of a combined treatment of insulin glargine and propylene glycol on the resolution of hyperketonemia and milk production in postpartum dairy cows

**DOI:** 10.3168/jdsc.2022-0228

**Published:** 2022-07-09

**Authors:** J. Denis-Robichaud, S. Buczinski, V. Fauteux, J. Dubuc

**Affiliations:** 1 Independent researcher, Amqui, Québec, Canada, G5J 2N5; 2 Faculté de Médecine Vétérinaire, Université de Montréal, 3200, rue Sicotte, Saint-Hyacinthe, Québec, Canada, J2S 2M2

## Abstract

Multiple treatments for hyperketonemia have been studied, and although propylene glycol has been the most constantly reported as successful in mitigating the effects of this condition, a recent study showed that a subpopulation of cows affected by hyperketonemia and hypoglycemia could benefit from a combined treatment of propylene glycol and insulin glargine. The objective of the present study was to assess the effect of this combination on the time-to-hyperketonemia resolution, and on milk production following the initial treatment. A randomized controlled trial was conducted on 2 commercial farms on which postpartum exams were conducted twice a week. Cows naturally affected by both hyperketonemia [β-hydroxybutyrate (BHB) ≥ 1.4 mmol/L] and hypoglycemia (glucose <2.2 mmol/L) were enrolled and randomly assigned to receive 2 mL of placebo (physiological saline) subcutaneously (PBO), or to receive 2 mL of insulin glargine (200 IU) subcutaneously (INS). Cows in both groups also received 300 g of propylene glycol orally for 3 d. Time-to-hyperketonemia resolution (return to BHB <1.4 mmol/L) and daily milk production following the initial treatment were compared between groups using Cox proportional hazard and mixed linear regression models, respectively. A total of 248 cows between 1 and 30 d in milk (median = 16) were enrolled in the trial from January 2018 to February 2022. Cows were of first (32.3%; n = 80/248), second (32.3%; n = 80/248), or third or greater (35.4%; n = 88/248) parity. The overall time-to-hyperketonemia resolution was not different between treatments, but there was a 2-way interaction between treatment group and parity. Primiparous cows had a shorter time-to-resolution in the INS group than in the PBO group; no difference was observed between treatment groups for older cows. The overall milk production of cows in the INS group was 3.4 kg higher than cows in the PBO group during the 14 d after enrollment. There was a 2-way interaction between treatment group and parity. Primiparous cows in the INS group produced 5.3 kg more milk daily than primiparous cows in the PBO group; no effect of treatment was found for older cows. These results suggest that cows affected simultaneously by hyperketonemia and hypoglycemia benefited from a combined treatment of propylene glycol and insulin glargine to improve subsequent milk production and that the effect primarily came from the positive effect in primiparous cows. Time-to-hyperketonemia resolution was also improved in primiparous cows but not in older cows.

## Highlights

• Cows were enrolled when simultaneously hyperketonemic and hypoglycemic.
• Treatment with propylene glycol and insulin glargine did not improve overall time-to-hyperketonemia resolution.
• Treatment with propylene glycol and insulin glargine improved overall milk production after enrollment.
• For both outcomes, there was a clear effect of treatment in primiparous cows but none in older cows.

## Graphical Abstract

**Figure:**
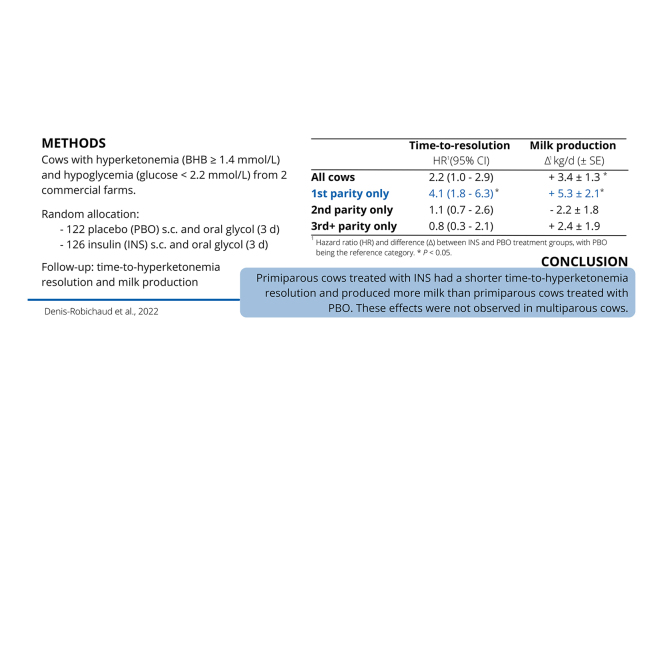
**Summary:** The objective of this study was to assess if a treatment of propylene glycol combined with insulin glargine for cows affected simultaneously by hyperketonemia and hypoglycemia would improve the time-to-hyperketonemia resolution and milk production after treatment. A total of 248 cows were randomly assigned to a propylene glycol and placebo treatment or a propylene glycol and insulin glargine treatment. Overall, there was a positive effect treatment for improving subsequent milk production in the 14 days following enrollment but there was no effect on time-to-hyperketonemia resolution. More specifically, primiparous cows treated with propylene glycol and insulin glargine had a shorter time-to-hyperketonemia resolution and produced more milk than primiparous cows treated with propylene glycol and the placebo; these effects were not found in older cows.

When dairy cows calve, the beginning of lactation challenges their metabolism and immunity, which results in a high-risk period for metabolic and infectious diseases. Although the prepartum period is of utmost importance to preventing peripartum problems (recently reviewed by Pascottini et al., 2020), postpartum diseases keep occurring in North American dairy herds. Hyperketonemia is defined as an elevated concentration of circulating ketone bodies such as BHB, and is one of the conditions of interest in the postpartum period as it has been associated with the occurrence of other diseases, low milk production, and subsequent reproductive problems (Duffield et al., 2009; Ospina et al., 2010a,b). In Québec (Canada), a herd-level study investigating the prevalence of hyperketonemia in the first 14 DIM, defined as a concentration of blood BHB ≥1.4 mmol/L, showed that it varied between 4 and 75%, with a median prevalence of 19% (Dubuc and Denis-Robichaud, 2017). A common treatment for this condition is propylene glycol (McArt et al., 2011, 2012; Capel et al., 2021), which was shown to have a positive effect on subsequent milk yield, resolution of hyperketonemia, displaced abomasum, reproduction, and culling (McArt et al., 2011, 2012). Additional treatments, usually combined with propylene glycol, have also been assessed including dextrose, glucocorticoids, B vitamins, and insulin glargine (Tatone et al., 2016; Gordon et al., 2017; Capel et al., 2021). Insulin glargine yielded interesting results in a multiherd randomized clinical trial where cows treated with propylene glycol and insulin glargine did not, overall, benefit from the treatment, but a subpopulation of hyperketonemic cows that were simultaneously hypoglycemic (blood glucose <2.2 mmol/L) produced 4.2 kg more milk per day than their counterparts that received a placebo treatment (Gordon et al., 2017). In the same study, hyperketonemic cows that were considered normoglycemic and that received insulin glargine produced 2.3 kg/d less than their counterparts that received a placebo. These surprising results raise the question of why some hyperketonemic cows would benefit from insulin glargine and others would not. The results reported by Gordon et al. (2017) were found while testing statistical model interactions and it remains unclear if these results can be repeated. Hypoglycemia has been shown to be present in about one-third of hyperketonemic cows, with a herd prevalence of simultaneous hypoglycemia and hyperketonemia during the first 14 DIM varying between 3 and 18% (median = 8%; Dubuc and Buczinski, 2018). Therefore, the objective of the present study was to quantify the effect of insulin glargine, used in combination with propylene glycol, on hyperketonemia resolution and subsequent milk production in cows affected simultaneously by hyperketonemia and hypoglycemia.

This randomized controlled trial was approved by the Animal Care Committee of the Université de Montréal (Rech-1747), and the REFLECT statement was used to report the findings (O'Connor et al., 2010). The unit of interest was the cow. Data collection on farms was performed between January 2018 and February 2022. The study was conducted in 2 commercial dairy herds located in the St-Hyacinthe area (QC, Canada) and selected by convenience for their location and implementation of standardized operating procedures for fresh cow surveillance and treatment. The herds both had 300 lactating cows, which were fed a TMR, housed in a freestall barn, and milked in a parlor 3 times a day. All fresh cows from these herds were examined twice a week (Tuesdays and Fridays) by an animal health technician during their first 30 d postpartum.

At each of these exams, a 1-mL blood sample was taken from the coccygeal vessels and blood BHB was immediately measured using a cow-side device validated for dairy cows (Precision Xtra; Abbott; Iwersen et al., 2009). When a cow had a BHB ≥1.4 mmol/L (hyperketonemia), the remainder of the blood sample was used to quantify blood glucose using the same cow-side device with strips validated for glucose measurements (Wittrock et al., 2013). Hyperketonemic cows with a blood glucose <2.2 mmol/L (hypoglycemia) were systematically enrolled in the current study and randomly assigned to 1 of 2 treatments. Treatment allocation was performed using a random number generator (Microsoft Excel, Microsoft Corporation) and treatments were balanced within groups of 10 cows. Cows in the control/placebo group (**PBO**) were treated once subcutaneously with 2 mL of placebo (physiological saline; 0.9% NaCl, Baxter Corporation). Cows in the insulin group (**INS**) were treated subcutaneously with 2 mL of insulin glargine (200 IU; Lantus, Sanofi-Aventis Canada). Cows in both groups were treated orally with 300 g of propylene glycol PO (300 mL; propylene glycol-P, Vetoquinol) for 3 d. Enrollment, treatment group allocation, and initial treatment were done by the technician, who was blinded to group allocation. All syringes (placebo or insulin) were prepared in advance by a blinded person (pharmacist) and all appeared identical. The farm manager in charge of performing daily treatments (oral propylene glycol) was also blinded to treatment group allocation.

Once cows were enrolled in the trial, their blood BHB was tested twice a week by the research technician until resolution (return to BHB <1.4 mmol/L), and daily milk production was recorded for 14 d after enrollment using farm equipment (GEA milking parlor; GEA Farm Technologies Canada Inc.). Cows could only be enrolled once in the trial.

A sample size of 40 cows per treatment group was estimated for identifying a difference of 3.8 kg (variance = 36) in subsequent daily milk production, with 95% confidence and 80% power (Dohoo et al., 2009), and accounting for a 5% loss to follow-up. This sample size estimation was based on the results published by Gordon et al. (2017). To properly quantify a probable interaction effect between parity group and treatment, a sample size of 80 cows (40 PBO and 40 INS) for each parity group (1, 2, 3+) was targeted (240 cows total; 120 per treatment group).

Statistical analyses were conducted in R (version 4.0.5; R Core Team, 2015; using the RStudio interface; version 1.3.1093). Descriptive statistics were computed for all cows and compared between treatment groups. Time-to-hyperketonemia resolution was defined as the period between enrollment and the next examination day for which BHB was <1.4 mmol/L. Herd clustering was treated as a fixed effect in all models. The effect of treatment group on time-to-resolution was assessed using Cox proportional hazards regression models ([*] coxph function, survival package; Therneau, 2021). The assumption of proportional hazards was validated. The effect of treatment on daily milk production during the 14 d following initial treatment was assessed using mixed linear regression models accounting for cows as a random intercept (repeated measures using unstructured covariance structure) and day of sampling as a fixed effect ([*] lmer function, lme4 package; Bates et al., 2015). Data from cows that died or were culled during the 14 d following the initial treatment were used until the day of removal from the herd in all models. For final models, marginal means and contrasts were estimated accounting for multiple comparisons ([*] emmeans function, emmeans package; Searle et al., 1980; Lenth et al., 2021). As this was a randomized controlled trial, no covariables were included in the models, but the 2 interactions between treatment and parity were assessed in all models as previous results identified parity as a modifier effect of treatment (Gordon et al., 2017). Significance was declared for *P* ≤ 0.05.

A total of 248 cows (122 in the PBO group; 126 in the INS group) were enrolled in the trial from January 2018 to February 2022. Cows were enrolled in the study between 1 and 30 DIM (median = 16; mean = 15.6) and were of first (32.3%; n = 80/248), second (32.3%; n = 80/248), or third or greater (35.4%; n = 88/248) parity. At enrollment, their blood BHB ranged from 1.4 to 6.2 mmol/L (median = 2.7; mean = 2.9), whereas their glucose ranged from 1.0 to 2.2 mmol/L (median = 1.8; mean = 1.9), and their daily milk production ranged from 12.5 to 59.7 kg (median = 38.7; mean = 38.2). Three cows were culled during the study period: PBO group (n = 2; 7 and 14 d after the initial treatment) and INS group (n = 1; 3 d after the initial treatment) due to injury (n = 1), mastitis (n = 1), and lameness (n = 1). Cows in the 2 treatment groups did not differ in season, parity, DIM, milk production, BHB at enrollment, or blood glucose at enrollment (Table 1). No adverse event occurred during the study.

**Table 1:** Descriptive statistics at enrollment of 248 cows affected simultaneously by hyperketonemia^1^ and hypoglycemia^2^ and enrolled in a randomized controlled trial comparing 2 treatments: physiological saline (PBO; 2 mL subcutaneously once) and 3 d of oral propylene propylene glycol (300 g/d); or insulin glargine (INS; 2 mL subcutaneously once; 200 IU) and 3 d of oral propylene propylene glycol (300 g/d)

Description at enrollment	Treatment		*P*-value^3^
	INS (n = 126)	PBO (n = 122)	
Season (n; %)			0.42
Winter (Jan to Mar)	38 (30.2)	43 (35.2)	
Spring (Apr to Jun)	32 (25.4)	32 (26.2)	
Summer (Jul to Sep)	32 (25.4)	29 (23.8)	
Fall (Oct to Dec)	24 (19.0)	16 (13.1)	
Parity (n; %)			0.71
First	40 (31.7)	40 (32.8)	
Second	40 (31.7)	40 (32.8)	
Third and greater	46 (36.6)	42 (34.4)	
DIM (d ± SD)			
All	16.2 ± 7.6	15.8 ± 8.4	0.51
First parity	15.8 ± 8.1	15.2 ± 8.9	0.68
Second parity	16.7 ± 8.3	16.3 ± 9.2	0.67
Third and greater	16.0 ± 7.9	15.7 ± 9.1	0.72
Daily milk production (kg ± SD)			
All	39.1 ± 10.5	39.3 ± 9.5	0.82
First parity	32.8 ± 11.4	32.5 ± 10.3	0.93
Second parity	42.9 ± 11.6	42.6 ± 9.9	0.87
Third and greater	40.0 ± 10.9	40.4 ± 10.4	0.91
Blood BHB (mmol/L ± SD)			
All	2.9 ± 1.2	2.9 ± 1.1	0.88
First parity	2.8 ± 1.5	3.0 ± 1.3	0.64
Second parity	2.9 ± 1.3	3.1 ± 1.4	0.71
Third and greater	3.0 ± 1.4	2.9 ± 1.5	0.85
Blood glucose (mmol/L ± SD)			
All	1.8 ± 0.4	1.8 ± 0.3	0.42
First parity	1.7 ± 0.6	1.8 ± 0.5	0.59
Second parity	1.8 ± 0.6	1.9 ± 0.5	0.62
Third and greater	1.8 ± 0.7	1.9 ± 0.6	0.68

1 Hyperketonemia was defined as blood BHB ≥1.4 mmol/L in the first 30 d postpartum.

2 Hypoglycemia was defined as blood glucose <2.2 mmol/L in the first 30 d postpartum.

3 *P*-values obtained from Pearson's chi-squared test for categorical variables, and linear regression models for continuous variables.

Overall time-to-hyperketonemia resolution was not different between treatment groups [hazard ratio (95% CI) = 2.2 (1.0–2.9); *P* = 0.07; Figure 1A], but there was a 2-way interaction between treatment and parity (*P* = 0.01). Primiparous cows in the INS group returned to blood BHB <1.4 mmol/L faster than primiparous cows in the PBO group [hazard ratio (95% CI) = 4.1 (1.8–6.3); *P* = 0.01], whereas no difference was observed between treatment groups for multiparous cows [second parity: hazard ratio (95% CI) = 1.1 (0.7–2.6); *P* = 0.65; third and greater parity: hazard ratio (95% CI) = 0.8 (0.3–2.1); *P* = 0.66; Figure 1B]. Herd was not associated (*P* = 0.88) with time-to-hyperketonemia resolution.

**Figure 1:**
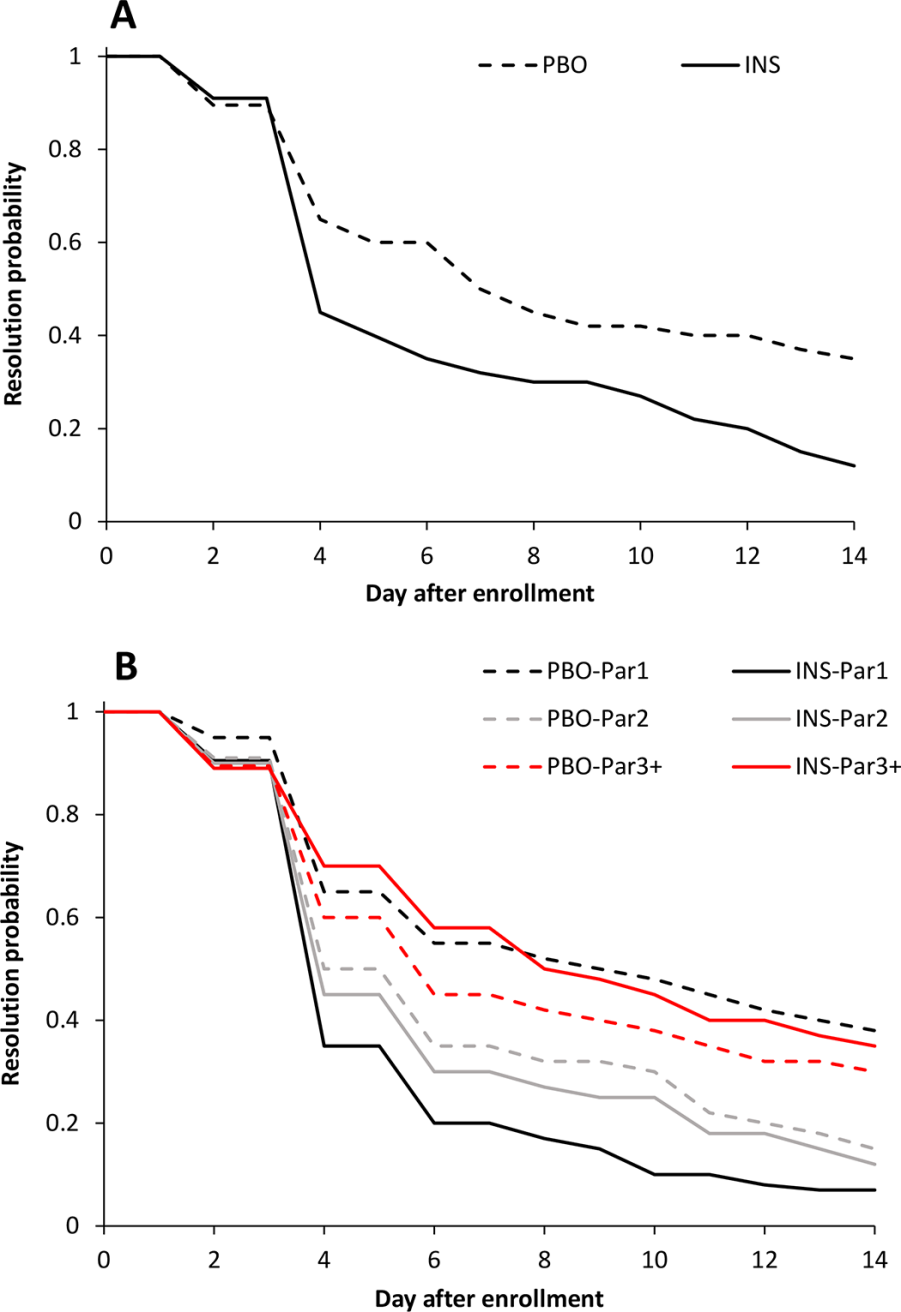
Effect of treatment on time-to-hyperketonemia resolution (return to blood BHB <1.4 mmol/L) following enrollment of 248 postpartum Holstein cows affected simultaneously by hyperketonemia and hypoglycemia. Cows were treated with 2 mL of physiological saline (PBO) subcutaneously and 3 d of propylene glycol (300 g/d) orally, or 2 mL (200 IU) of insulin glargine (INS) subcutaneously and 3 d of oral propylene glycol (300 g/d) orally. Panel A includes the overall data (hazard ratio = 2.2; *P* = 0.07), and panel B stratifies data by parity groups (Par1: hazard ratio = 4.1, *P* = 0.01; Par2: hazard ratio = 1.1, *P* = 0.65; Par3+: hazard ratio = 0.8, *P* = 0.66).

Daily milk production during the 14 d following initial treatment was 3.4 kg (SE = 1.3) higher for cows in the INS group compared with the PBO group [marginal means (95% CI): PBO = 42.3 (41.1–43.7) kg; INS = 45.7 (43.4–47.5) kg; *P* = 0.05; Figure 2A]. There was, however, a modifier effect by parity (*P* = 0.01) showing that the positive impact of insulin glargine was mainly present in primiparous cows (Figure 2B). As such, primiparous cows in the INS group produced 5.3 kg (SE = 2.1) more milk daily than primiparous cows in the PBO group [marginal means (95% CI): PBO = 35.8 (32.7–39.0) kg; INS = 41.1 (37.0–45.0) kg; *P* = 0.01]. For second parity cows, those in the INS group produced 2.2 kg (SE = 1.8) less milk daily than cows in the PBO group [marginal means (95% CI): PBO = 45.7 (41.1–49.5) kg, INS = 43.5 (40.2–45.6) kg; *P* = 0.21]. For cows of third or greater parity, cows in the INS group produced 2.4 kg (SE = 1.9) more milk daily than cows in the PBO group [marginal means (95% CI): PBO = 43.5 (41.4–45.7) kg; INS = 45.9 (43.9–48.00) kg; *P* = 0.13]. Herd was not associated (*P* = 0.71) with milk production.

**Figure 2:**
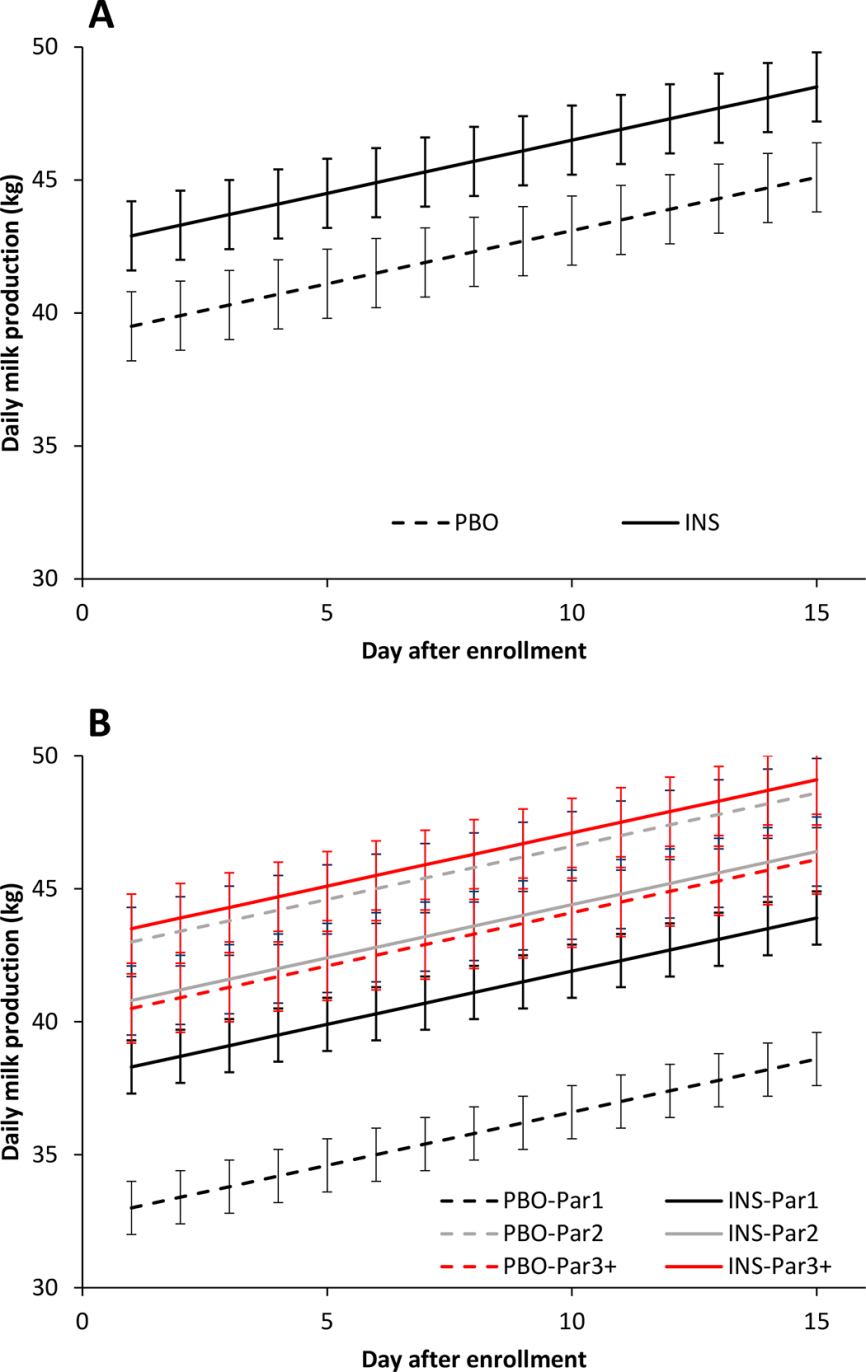
Effect of treatment (± SE) after enrollment on subsequent milk production in 248 postpartum Holstein cows affected simultaneously by hyperketonemia and hypoglycemia. Cows were treated with 2 mL of physiological saline (PBO) subcutaneously and 3 d of propylene glycol (300 g/d) orally, or 2 mL (200 IU) of insulin glargine (INS) subcutaneously and 3 d of oral propylene glycol (300 g/d) orally. Panel A includes the overall data (+3.4 kg, *P* = 0.05), and panel B stratifies data by parity groups (Par1: +5.3 kg, *P* = 0.01; Par2: −2.2 kg, *P* = 0.21; Par3+: + 0.13 kg, *P* = 0.13).

Hyperketonemic cows with simultaneous hypoglycemia benefited from treatment differently than expected at the start of the study. Overall, cows in the INS group did not benefit from treatment to reduce time-to-hyperketonemia resolution delay compared with cows in the PBO group. However, cows in the INS group had higher subsequent daily milk production than cows in the PBO group. There was, however, a modifying effect of parity for both outcomes [as reported by Gordon et al. (2017) for milk production]. As such, only primiparous cows benefited from the insulin glargine treatment for reducing time-to hyperketonemia resolution delay and for improving subsequent milk production. Other parities did not benefit sufficiently from the insulin glargine treatment to obtain statistically significant results. Regarding the time-to-hyperketonemia resolution delay, these was no numerical difference between treatment groups for parities 2 or greater. Similar to the results reported by Gordon et al. (2017), cows of second parity had a detrimental numerical effect on milk production (−2.2 kg/d) of the insulin glargine treatment. The numerical difference between treatments in cows of parity 3 or greater was positive (+2.4 kg/d) but not sufficient to be statistically significant.

While the current trial was developed based on findings of Gordon et al. (2017), there were multiple differences between the cow population and study design of these 2 trials, which make comparisons difficult. A similarity was that insulin glargine treatment in primiparous cows resulted in an increase in milk production for hyperketonemic cows, but this was regardless of their glycemic status (Gordon et al., 2017). As normoglycemic cows were not included in the present study, it is unclear if we would have found similar results. Moreover, the study by Gordon et al. (2017) showed, using stratified data, that hyperketonemic cows of third parity or greater with simultaneous hypoglycemia were the subpopulation benefiting the most from the insulin glargine treatment, which was not the case in the present study. Although the current study identified hyperketonemic primiparous cows with simultaneous hypoglycemia as a group that benefits the most from a treatment of propylene glycol combined with insulin glargine, it remains unclear if these results would be generalizable to all farms, considering the contradicting results with Gordon et al. (2017). An aspect that could influence our results is the farm management of replacement animals. Indeed, the current study was conducted in 2 herds and their management likely influenced the metabolic health of their cows. It has been shown that glucose-dependent insulin secretion is decreased in postpartum cows (Weber et al., 2016), and tissues are less responsive to insulin to spare glucose for milk production, suppressing fat mobilization, and slowing down ketone body production (Hayirli, 2006). It is possible that cows affected simultaneously by hyperketonemia and hypoglycemia would benefit from slowing down fat mobilization for a short period, as they are likely hypoinsulinemic and have effective glucogenic pathways, but insufficient glucose precursors to meet the high demand (Holtenius and Holtenius, 1996). The consequences of hyperketonemia in hypoglycemic cows could consequently be alleviated with exogenous insulin in combination with a source of glucose. It is unclear, however, why primiparous or multiparous cows only would be more likely to benefit from this treatment. It could be that their management, and consequently their fat reserve and milk production in early lactation, differed among farms. Further studies on the physiological effects of the treatment (propylene glycol and insulin glargine) in cows of different parities could help elucidate our findings. This could be relevant as it was quantified that roughly one-third of hyperketonemic cows also have simultaneous hypoglycemia (Dubuc and Buczinski, 2018). Thus, identifying a treatment that could help this subpopulation of cows would be relevant for improving the animals' health and production.

Before using insulin glargine on dairy farms, it is important to consider the fact that this drug is approved for human use. Depending on the legislation of each country, its extra-label use in food-producing animals might be allowed or restricted. Thus, caution should be used.

The conclusion of the present study is that in the 2 studied herds, cows affected simultaneously by hyperketonemia and hypoglycemia did benefit from a combined treatment of propylene glycol and insulin glargine to improve subsequent milk production and that effect primarily came from the positive effect in primiparous cows. On the other hand, such treatment did not have an overall impact on time-to-hyperketonemia resolution except for primiparous cows that benefited from it.
